# Phase I study of low-dose metronomic temozolomide for recurrent malignant gliomas

**DOI:** 10.1186/s12885-016-2945-2

**Published:** 2016-11-22

**Authors:** Eric T. Wong, Joshua Timmons, Amy Callahan, Lauren O’Loughlin, Bridget Giarusso, David C. Alsop

**Affiliations:** 1 Brain Tumor Center & Neuro-Oncology Unit, Department of Neurology, Beth Israel Deaconess Medical Center, Harvard Medical School, 330 Brookline Avenue, Boston, Massachusetts 02215 USA; 2 MRI Research, Department of Radiology, Beth Israel Deaconess Medical Center, Harvard Medical School, 330 Brookline Avenue, Boston, Massachusetts 02215 USA

**Keywords:** Metronomic temozolomide, Recurrent glioma, Arterial spin labeling, Matrix metalloproteinase, Interleukin

## Abstract

**Background:**

The treatment goal for recurrent malignant gliomas centers on disease stabilization while minimizing therapy-related side effects. Metronomic dosing of cytotoxic chemotherapy has emerged as a promising option to achieve this objective.

**Methods:**

This phase I study was performed using metronomic temozolomide (mTMZ) at 25 or 50 mg/m^2^/day continuously in 42-day cycles. Correlative studies were incorporated using arterial spin labeling MRI to assess tumor blood flow, analysis of matrix metalloproteinase-2 (MMP-2) and MMP-9 activities in the cerebrospinal fluid (CSF) as surrogates for tumor angiogenesis and invasion, as well as determination of CSF soluble interleukin-2 receptor alpha (sIL-2Rα) levels as a marker of immune modulation.

**Results:**

Nine subjects were enrolled and toxicity consisted of primarily grade 1 or 2 hematological and gastrointestinal side effects; only one patient had a grade 3 elevated liver enzyme level that was reversible. Tumor blood flow was variable across subjects and time, with two experiencing a transient increase before a decrease to below baseline level while one exhibited a gradual drop in blood flow over time. MMP-2 activity correlated with overall survival but not with progression free survival, while MMP-9 activity did not correlate with either outcome parameters. Baseline CSF sIL-2Rα level was inversely correlated with time from initial diagnosis to first progression, suggesting that subjects with higher sIL-2Rα may have more aggressive disease. But they lived longer when treated with mTMZ, probably due to drug-related changes in T-cell constituency.

**Conclusions:**

mTMZ possesses efficacy against recurrent malignant gliomas by altering blood flow, slowing invasion and modulating antitumor immune function.

## Background

Patients with recurrent malignant glioma have poor prognosis. Their respective median progression free survival (PFS) and overall survival (OS) are 10 and 30 weeks, while the 6-month PFS is 15% [1]. Although bevacizumab and tumor treating fields are currently approved treatments, patient tumors can still progress despite active interventions [2–4]. In particular, patients who failed bevacizumab almost always exhibit diffusely invasive disease within the brain. Their respective PFS and OS are 9 and 23 weeks, and their 6-month PFS is 0% [5]. Therefore, new strategies that can halt further progression of recurrent gliomas and neurologic deficits are needed for this population.

Temozolomide (TMZ) is an alkylating chemotherapy prodrug with activity against recurrent malignant gliomas [6, 7]. It undergoes spontaneous aqueous conversion to 5-(3-dimethyl-1-triazenyl)imidazole-4-carboxamide (MTIC) which then produces diazomethane capable of alkylating the O^6^-position of guanine in DNA [8]. The recommended dosing schedule of 150–200 mg/m^2^/day for 5 days is based on a typical phase I dose escalation study with this as the maximum tolerated dose, and myelosuppression was the dose-limiting toxicity [8]. The rationale behind maximum tolerated dose is to use the highest concentration of chemotherapy to directly kill tumor cells, while the patient can still withstand side effects. Unfortunately, this approach may interfere with other important antitumor mechanisms of TMZ. Metronomic temozolomide (mTMZ) schedule consists of a significantly lower daily dose but at a greater frequency of administration, typically at 25 or 50 mg/m^2^/day on a continuous basis. The biological effect of this schedule is likely to be different from that of conventional dosing, and mTMZ has been shown to selectively deplete CD4^+^CD25^+^Foxp3^+^ regulatory T cells (Tregs), which play important roles in supporting immunosuppression within the microenvironment of malignant gliomas [9, 10].

Similar anti-tumor benefits have been observed in metronomic cyclophosphamide, which also alkylates DNA but requires metabolism by the liver for its conversion to phosphamide mustard that causes DNA cross-linking. Metronomic cyclophosphamide has been demonstrated to exert an antiangiogenic effect. This is thought to be a result from the heightened sensitivity of endothelial cells, relative to tumor cells, to the cytotoxic effect of chemotherapies while side effects on fast dividing hematopoietic and intestinal cells are minimized [11, 12]. Cyclophosphamide also depletes Tregs that play immunosuppressive roles within tumors, and it has been used to facilitate adoptive immunotherapy [13–15]. In addition, dacarbazine, which like TMZ produces MTIC as its active metabolic intermediate, has been shown to upregulate natural killer group 2D (NKG2D) ligands on melanoma cells and to sensitize them for clearance by natural killer (NK) and CD8^+^ T cells in mouse models [16]. Treatment of tumor cells with DNA-alkylating agents can also result in their secretion of high-mobility group box 1 cytokine, which stimulates the migration and activation of cytotoxic effector immune cells [17]. Therefore, TMZ has the potential to promote immunostimulatory antitumor effects and it may achieve this at below the standard-of-care doses, which are often derived from dose escalation studies based on the maximum tolerated off-target side effects rather than efficacy.

We report here a phase 1 study of mTMZ in patients with recurrent malignant glioma, in conjunction with an exploratory analysis of tumor blood flow using arterial spin labeling magnetic resonance imaging (MRI). We also measured levels of soluble interleukin-2 receptor alpha (sIL-2Rα) and the activated isoforms of matrix metalloproteinases (MMPs) from patient cerebrospinal fluid (CSF) to determine whether these potential biomarkers of immunogenicity and angiogenesis/invasion correlate with patient outcome.

## Methods

### Study design and patient eligibility

This study was conducted between July 2006 and September 2011 after obtaining ethics approval from the Institutional Review Board at Beth Israel Deaconess Medical Center. All participants provided written informed consent for study treatment and for publication of trial outcome. Subjects were stratified according to a 3 × 3 factorial design based on the histological diagnosis of either grade IV glioblastoma or grade III malignant glioma, as well as by the dosage of mTMZ either at 25 or 50 mg/m^2^/day taken continuously for 42 days in a cycle. Subjects were enrolled if they had (i) age ≥18, (ii) recurrent high-grade glioma histologically confirmed either at initial diagnosis or at recurrence, (iii) conventional involved-field radiotherapy, (iv) Karnofsky performance score ≥60, (v) bi-dimensionally measureable disease, (vi) no concurrent malignancy other than basal or squamous cell carcinoma of the skin, or carcinoma in situ of the cervix, (vii) stable dose of corticosteroid for ≥3 days, and (viii) adequate hematologic, renal and liver functions. Subjects were excluded if they had (i) multifocal glioma, gliomatosis cerebri, low-grade glioma, or leptomeningeal spread of the malignant glioma, (ii) difficulty undergoing MRI scanning, (iii) chemotherapy, immunotherapy, or biologic therapy within 4 weeks prior to study, (iv) poor recovery from prior therapies, (v) poor medical risks, (vi) difficulty recovering from any effect of major surgery, (vii) requirement for P450 hepatic enzyme inducing anticonvulsant, or (viii) HIV or acquired immunodeficiency syndrome. Treatment was continued until disease progression as defined by Macdonald’s criteria [18] or withdrawal from the trial. Clinical examination, conventional gadolinium-enhanced head MRI with arterial spin labeling sequence [19], and lumbar punctures were performed once before the first cycle and after each subsequent cycle.

### Assessment of safety and treatment outcome

Adverse events were recorded from subjects at baseline and during follow up in the trial period. Severity was graded according to the Common Toxicity Criteria version 3.0 and attribution was made to the study medication.

At the end of each 6-week mTMZ cycle, assessment for response or progression was made using gadolinium-enhanced T1-weighted images on MRI. Bi-dimensional tumor size was measured according to the Macdonald’s criteria [18].

### Correlative studies

Blood flow into the tumor was measured by arterial spin labeling during acquisition of anatomic MRI images in an effort to characterize the vascular effect of mTMZ. This technique was previously described in detail [19]. In brief, it utilizes repetitively pulsed radiofrequency and magnetic gradient fields to achieve continuous inversion of water. Acquisition was performed with a 1.5 s delay after labeling to allow the labeled blood to reach the microvasculature. Unlike contrast based perfusion studies, arterial spin labeling specifically uses tagged water to measure blood flow. Since water is freely diffusible across the vasculature, arterial spin labeling allows for an accurate quantitative assessment of blood flow that is independent of vascular permeability.

Blood flow from arterial spin labeling images was quantified as described by Jarnum et al. [20]. A region of interest (ROI) was drawn that contained the malignant glioma based on post-gadolinium T1-weighted images. The average blood flow, in absolute cc/g•min, was obtained by computing the mean value across all voxels within that ROI. In addition, a blood flow ratio was calculated based on the ROI of the tumor to a corresponding ROI in the contralateral brain to allow for comparison of the blood flow data across multiple scans obtained over time.

Enzyme-linked immunosorbent assay (ELISA) was performed on the CSF obtained from our subjects. CSF was collected at baseline and at the end of each metronomic cycle, stored at −80° C, and then thawed for batched analysis. DuoSet ELISA kits DY223, DY902, and DY911 were obtained from R&D for determination of sIL-2Rα, activated MMP-2 (aMMP-2) and activated MMP-9 (aMMP-9) levels, respectively.

### Statistics

PFS and OS curves were plotted according to the Kaplan-Meier method [21]. The strength of correlation between blood flow and clinical outcome, as well as between cerebrospinal fluid biomarkers and clinical outcome, was evaluated by linear regression. Significance was computed and plotted using Graphpad Prism 6 software. Fold change from baseline, if positive, was reported as the final blood flow ratio divided by the initial blood flow ratio minus 1. If negative, fold change was reported as the negative reciprocal of the final blood flow ratio divided by the initial blood flow ratio minus 1.

## Results

The demographic characteristics of the 9 subjects (6 with glioblastomas and 3 with anaplastic gliomas) entered into the study are listed in Table 1. Their median age was 64 (range 26–82) years and their median KPS was 70 (range 60–90). Because protocol accrual began in 2006 and ended in 2011, all subjects had been treated with the standard-of-care radiation with concomitant daily TMZ at the time of their initial diagnosis. However, the number of cycles of post-radiotherapy adjuvant TMZ received was variable, ranging from none to 20 completed cycles prior to enrollment. One subject with glioblastoma signed consent for the protocol but did not receive mTMZ at 25 mg/m^2^/day because of rapid clinical deterioration. Another subject with glioblastoma underwent one cycle of mTMZ treatment at 25 mg/m^2^/day. Four subjects received 50 mg/m^2^/day of mTMZ for 1, 2, 5 and 6 cycles. Two subjects with anaplastic gliomas (one small cell anaplastic astrocytoma and one anaplastic oligodendroglioma) received 2 and 19+ cycles of mTMZ at 25 mg/m^2^/day, while a third with anaplastic glioma completed 8+ cycles at 50 mg/m^2^/day.

**Table 1:** Patient characteristics and outcomes

No	Adjuvant TMZ cycles	mTMZ cycles	Histology	KPS score	Metronomic dosage (mg/m^2^/d)	Diagnosis to first recurrence (months)	Progression free survival (months)	Overall survival (months)
1	1	2	Glioblastoma	70	50	2.9	3.1	71.0
2	0	0	Glioblastoma	90	25	2.4	N/A	11.0
3	1	2	Small cell anaplastic astrocytoma	70	25	2.7	7.1	7.1
4	20	1	Glioblastoma	70	25	23.3	1.5	9.7
5	2	6	Glioblastoma	70	50	5.8	9.9	12.7
6	0	19	Anaplastic ogliodendroglioma	90	25	128.6	>107	Alive
7	13	1	Glioblastoma	70	50	18.1	1.9	12.6
8	1	5	Glioblastoma	60	50	3.6	11.4	14.4
9	0	8	Anaplastic glioma	70	50	120.0	22.9	33.7

Baseline characteristics and outcomes among subjects treated with mTMZ

### Safety and toxicity

mTMZ was well tolerated and, as expected, the most frequent adverse events were hematological in nature (Table 2). Grade 1 or 2 leukopenia and lymphopenia occurred in 2 subjects while anemia, neutropenia and thrombocytopenia were observed in 1 subject, but none experienced grade 3 or 4 hematological toxicity. Gastrointestinal side effects occurred in 3 subjects, with one experienced grade 3 elevation of liver enzyme that was resolved after discontinuation of mTMZ. Two additional subjects had grade 1 liver dysfunction. Additional minor side effects included thrush, zoster eruption and petechial rash, which were all of grade 1 severity.

**Table 2:** Adverse events from mTMZ that were tabulated during the study period

Adverse events	Severity number of patients (%)	
	Grade 1 & 2	Grade 3 & 4
Hematological		
Anemia	1 (11%)	0
Leukopenia	2 (22%)	0
Lymphopenia	2 (22%)	0
Neutropenia	1 (11%)	0
Thrombocytopenia	1 (11%)	0
Fatigue	1 (11%)	0
Gastrointestinal		
Increased alkaline phosphatase	0	0
Increased ALT	1 (11%)	1 (11%)
Increased AST	1 ( 11%)	0
Infection		
Thrush	1 (11%)	0
Zoster eruption	1 (11%)	0
Skin		
Petechial rash	1 (11%)	0

### Outcome analysis

**Fig. 1:**
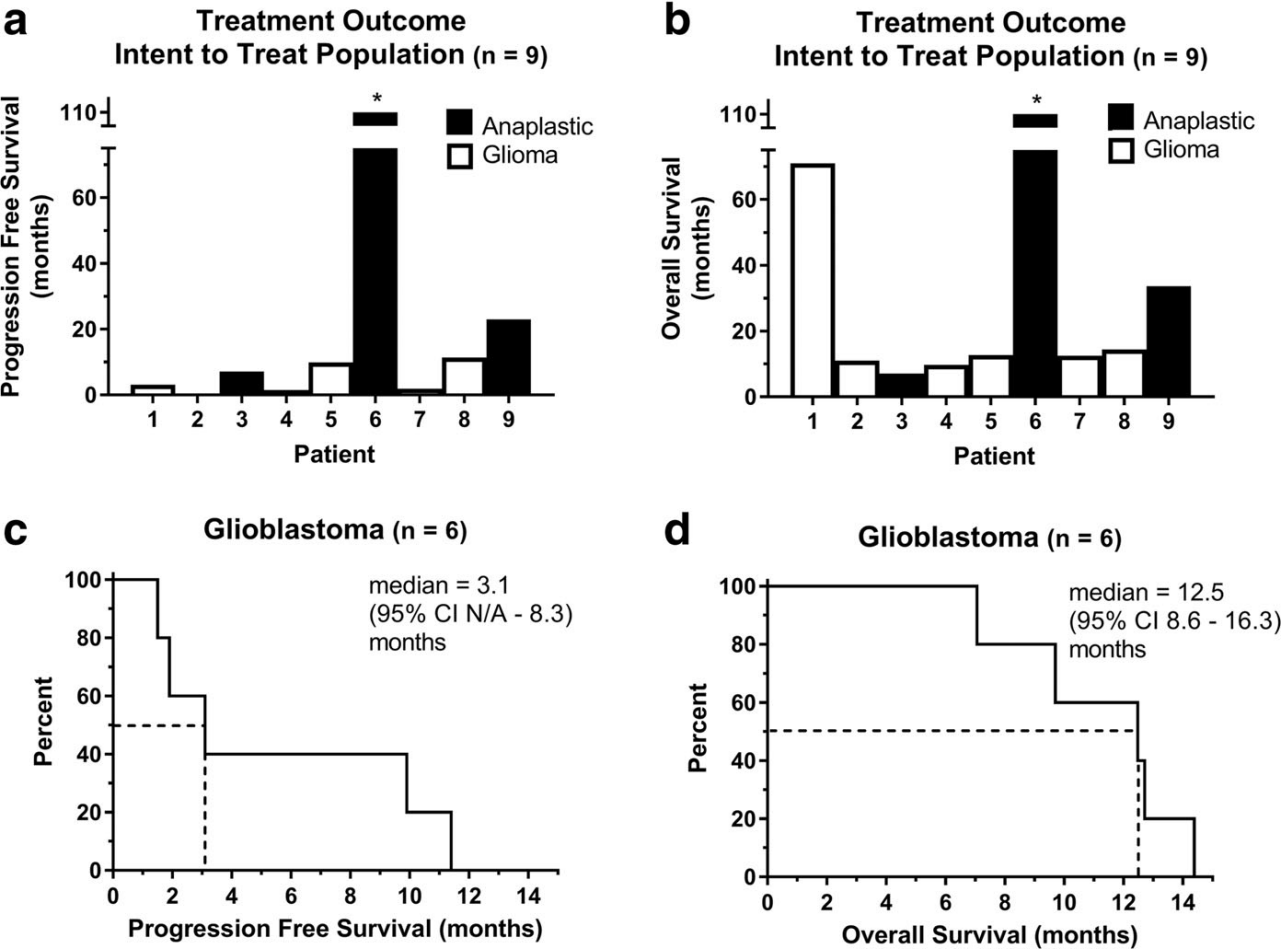
Treatment outcomes from mTMZ. Subjects with anaplastic glioma (*black*) and glioblastoma (*white*) and their individual (**a**) PFS and (**b**) OS are displayed individually. Six of 9 (67%) subjects had glioblastoma and their (**c**) PFS was 3.1 (95% CI N/A - 8.3) months and (**d**) OS was 12.5 (95% CI 8.6–16.3) months

The median number of mTMZ cycles received within the study group was 2 (range 0-19+), and the median time from initial diagnosis to first recurrence was 5.8 (range 2.4–128.6) months (Table 1). The number of prior adjuvant TMZ cycles received does not appear to correlate with the number of mTMZ cycles (Spearman correlation = −0.3914, *p* = 0.3053). The median progression free survival was 8.5 (range 1.5–153.0+) months and the median overall survival was 12.7 (range 7.1–153.0) months (Fig. 1a & 1b ). Because 6 of 9 subjects (67%) had recurrent glioblastoma, and they compromise the largest subgroup in our cohort with similar histological characteristics, we decided to combine their outcomes to estimate the benefit of mTMZ treatment. Their median progression free survival was 3.1 (95% CI N/A-8.3) months and their overall survival was 12.5 (95% CI 8.6–16.3) months (Fig. 1c & 1d).

### Correlative studies

**Fig. 2:**
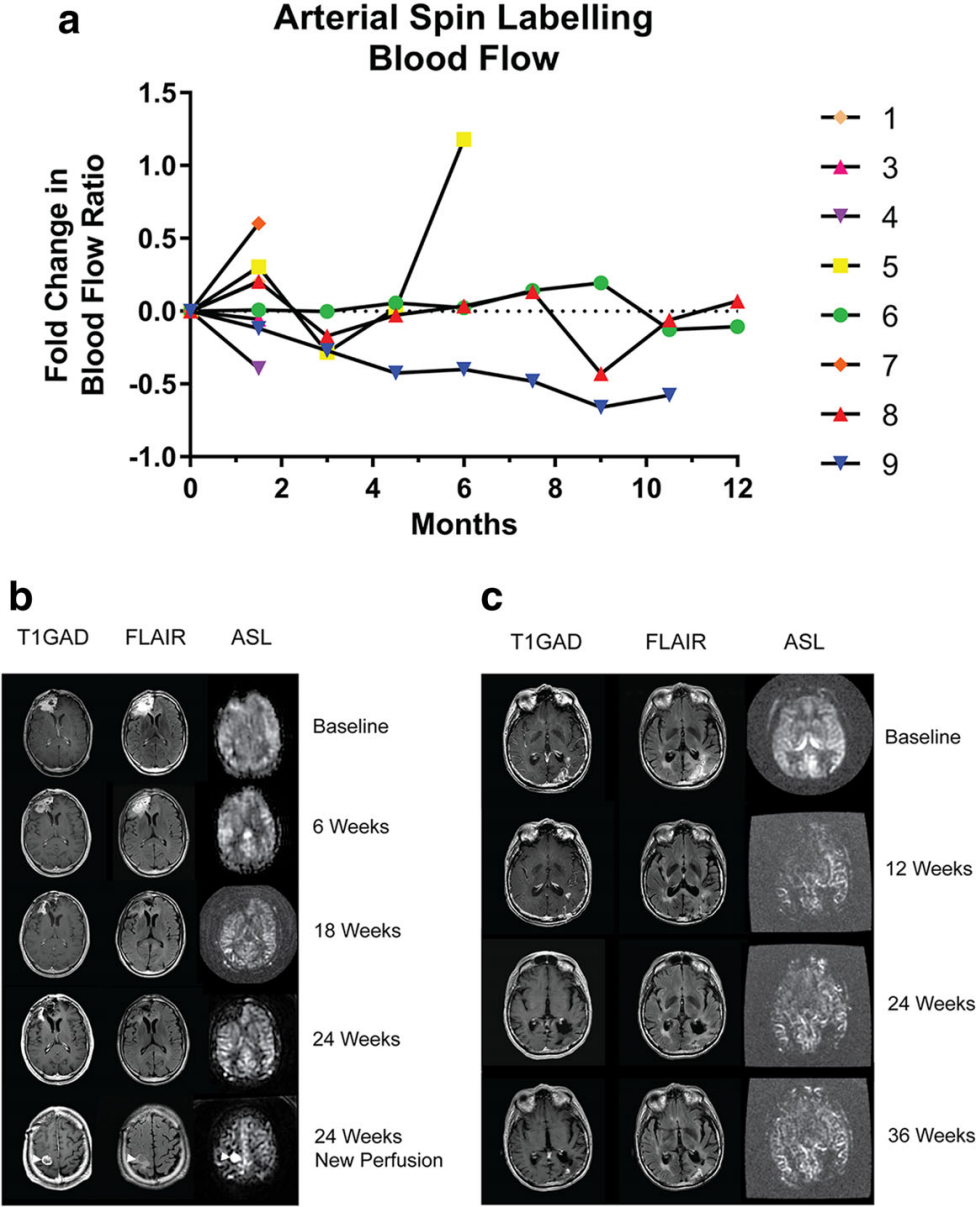
ASL-based blood flow is altered by mTMZ. **a** Spider plot of ASL blood flow in individual subjects. **b** Subject 5 had an initial increase in the normalized blood flow ratio from 0.70 at baseline to 0.92 at 6 weeks, followed by a decrease to 0.51 at 12 weeks and subsequently two successive increases to 0.72 and 1.53 at 18 and 24 weeks, respectively, as a result of a new focus of tumor in the ipsilateral brain (*arrowhead*). **c** Subject 9 had a gradual and sustained decrease of more than 50% in the blood flow ratio over time, from 0.91 at baseline to 0.39 at 54 weeks

Two types of correlative analysis were performed to help elucidate the antiangiogenesis and antitumor effects of mTMZ. The first type consisted of arterial spin labeling blood flow studies obtained serially in subjects at 6-week intervals during anatomic MRI scanning. It is noteworthy that there was marked variability in blood flow over time in our cohort during treatment (Fig. 2a), with two subjects initially experiencing a slight increase before a decrease was observed while two others had a gradual but consistent decline in blood flow. In particular, subject 5 had an increase in the normalized blood flow ratio from 0.70 at baseline to 0.92 at 6 weeks, followed by a decrease to 0.51 at 12 weeks and subsequently two successive increases to 0.72 and 1.53 at 18 and 24 weeks, respectively, due to a new focus of tumor focus in the ipsilateral brain (Fig. 2b). Furthermore, subject 9 had a gradual and sustained decrease of more than 50% in the blood flow ratio over time, from 0.91 at baseline to 0.39 at 54 weeks (Fig. 2c). These fluctuations in blood flow could be a result of alterations in the vascular physiology of the tumor, mTMZ treatment-induced changes in blood flow, or a combination of both.

Additional analyses were performed to explore the relationship between tumor blood flow and patient outcome using (i) the baseline blood flow ratio as well as (ii) the change in the blood flow ratio between baseline and the first set of data (Table 3). There was no correlation between baseline blood flow ratio and PFS (*r* ^2^ = 0.2479, *p* = 0.3933), baseline blood flow ratio and OS (*r* ^2^ = 0.2829, *p* = 0.2774), initial change in blood flow ratio and PFS (*r* ^2^ = 0.1306, *p* = 0.5502), or initial change in blood flow ratio and OS (*r* ^2^ = 0.0312, *p* = 0.7762). Collectively, the highly variable blood flow characteristics in the tumor and our small patient sample size preclude any reasonable statistical analysis. However, we can still observe qualitative changes using arterial spin labeling and in particular those who stayed on therapy longest showed stable to decreasing blood flow in the tumor over time.

**Table 3:** Correlative biomarkers in subjects treated with mTMZ.  Tumor blood flow was measured by arterial spin labeling (ASL) MRI while CSF levels of aMMP-2, aMMP-9 and sIL-2α were measured by ELISA

No	Number of ASL scans	Tumor blood flow			aMMP-2		aMMP-9		sIL-2Rα	
		Blood flow average (cc/g · min, across all time points)	Blood flow ratio (Baseline)	Blood flow ratio (Mean, across all time points)	Baseline (ng/mL)	Mean (ng/mL)	Baseline (pg/mL)	Mean (pg/mL)	Baseline (pg/mL)	Mean (pg/mL)
1	2	11.7	0.98	1.08						
2	2	42.4	0.99	0.99						
3	2	26.0	1.35	1.31						
4	2	21.8	0.80	0.64	13.7	7.0	13.2	11.0	0	0
5	5	23.4	0.70	0.88	13.9	13.4	5.7	7.2	11.3	9.7
6	18	42.2	0.92	0.90	16.7	16.7	6.6	7.3	19.5	18.1
7	2	46.3	0.68	0.88	9.7	12.5	5.9	9.2	0	3.8
8	9	17.1	0.59	0.58	13.9	15.1	11.9	8.4	18.1	21.9
9	7	13.8	0.91	0.58	14.0	11.2	4.0	3.8	0	2.5

**Fig. 3:**
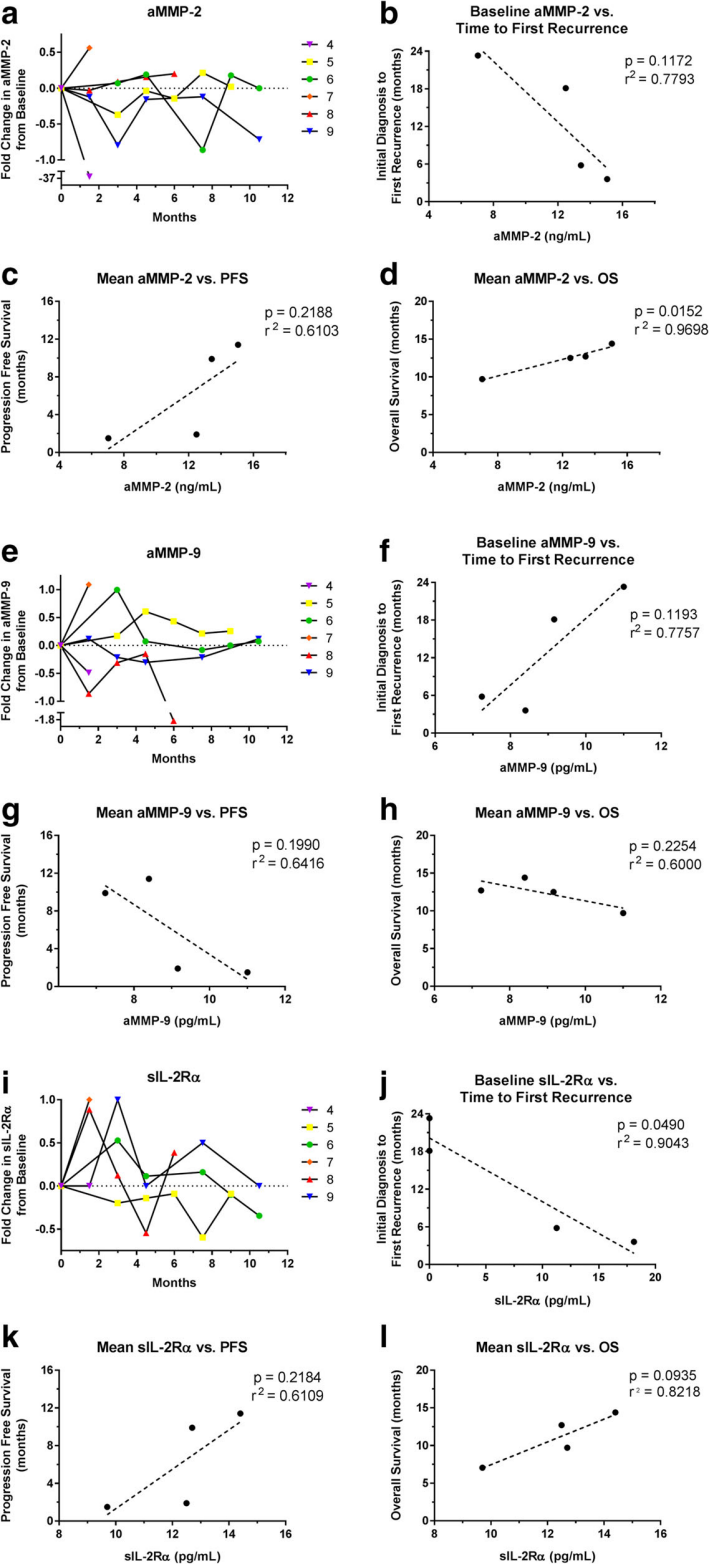
Correlative analyses of MMP-2, MMP-9 and sIL-2Rα in the CSF. (**a** to **d**) Activated MMP-2 has a bias toward lower levels when compared to baseline during treatment with mTMZ. The mean MMP-2 level has a direct correlation with OS but not PFS. (**e** to **h**) The level of activated MMP-9 is highly variable during treatment with mTMZ, but there was no correlation with OS or PFS. (**i** to **l**) sIL-2Rα levels are also highly variable among individual patients. Baseline sIL-2Rα level has an inverse correlation with time from initial diagnosis to first recurrence. There is a trend for correlation between increased sIL-2Rα level and OS but not PFS

CSF biomarkers relevant to the biological effects of mTMZ were also investigated. Specifically, MMP-2 and MMP-9 are activated during angiogenesis and glioma invasion, and both of these enzymes can be measured in the CSF. Indeed, our ELISA analyzed showed a bias toward lower levels of aMMP-2 compared to baseline as subjects were treated with mTMZ over time (Fig. 3a), while aMMP-9 levels remained highly variable in the CSF despite treatment (Fig. 3e). Furthermore, aMMP-2 directly correlated with OS (*r* ^2^ = 0.9698, *p* = 0.0152) (Fig. 3d) but not PFS (*r* ^2^ = 0.6103, *p* = 0.2188) (Fig. 3c), while aMMP-9 did not correlate with OS (*r* ^2^ = 0.6000, *p* = 0.2254) (Fig. 3h) or PFS (*r* ^2^ = 0.6416, *p* = 0.1990) (Fig. 3g). Baseline aMMP-2 (Fig. 3b) and aMMP-9 (Fig. 3f) did not correlate with time to first recurrence of the malignant glioma.

Previous studies have showed that metronomic dosing of TMZ can reduce the ratio of Treg/CD4^+^ cells whereas higher doses do not, and this reduction in Tregs could potentially reverse immunosuppression within the tumor microenvironment [9]. To investigate this aspect of mTMZ mechanism, we quantified the CSF levels of sIL-2Rα (also known as sCD25), which is known to counteract immune system activation in cancer patients and high levels of this biomarker in the serum have been correlated with poor survival [22–24]. Among our cohort with recurrent malignant gliomas, there was high variability in the levels of CSF sIL-2Rα (Fig. 3i) and high levels correlated with a shorter time from initial diagnosis to first recurrence (*r* ^2^ = 0.9043, *p* = 0.0490) (Fig. 3j). Notably, the two subjects with elevated levels of sIL-2Rα had the longest PFS (9.9 and 11.4 months) while the other two with undetectable levels possessed the shortest PFS (1.5 and 1.9 months). There was a trend for correlation between sIL-2Rα and OS (*r* ^2^ = 0.8218, *p* = 0.0935) (Fig. 3l) but not between sIL-2Rα and PFS (*r* ^2^ = 0.6109, *p* = 0.2184) (Fig. 3k).

## Discussion

Unlike the conventional schedule of TMZ at 150–200 mg/m^2^/day for 5 days, mTMZ is typically given continuously at a dose of 25 to 50 mg/m^2^/day. Such lower daily dosage may not be myelotoxic enough to cause significant leukopenia or thrombocytopenia while retaining antitumor efficacy and, when given over a longer period of time, the cumulative dose from mTMZ could be higher than the dose from conventional schedule. In the current study, TMZ given in metronomic doses was well tolerated by our subjects with recurrent malignant gliomas. The side effects observed were primarily hematological and gastrointestinal in nature, and nearly all of them were in the grade 1 or 2 severity category. This is consistent with findings in past phase II trials and retrospective series where others observed mild lymphopenia, neutropenia, thrombocytopenia and liver enzyme elevation [25–27].

Chronic daily dosing of cytotoxic chemotherapies have been in use as salvage treatment in oncology. Fulton et al. [28] reported the use of metronomic oral etoposide for recurrent malignant gliomas and noted an objective response rate of 18% (8 of 46 patients) and a median time to tumor progression of 8.8 weeks, while side effects consisted of manageable neutropenia and thrombocytopenia. Compared to pulsed intravenous administration of etoposide, metronomic oral etoposide has similar or even better bioavailability [29]. In addition, daily capecitabine is indicated for metastatic colon cancer and taxane-refractory breast cancer [30, 31]. Compared to intravenous 5-fluorouracil and leucovorin, capecitabine has less frequent hematological toxicity but more hepatic enzyme elevation, probably due to its first pass in the liver when taken orally [31].

Multiple mechanisms likely contribute to the antitumor efficacy of mTMZ. Our trial is the first to incorporate both neuroimaging and CSF correlative studies to help elucidate the underlying antitumor mechanisms of mTMZ. Arterial spin labeling MRI was used to measure blood flow at the site of disease and elsewhere in the brain. It is important to note that our current method of visualization of brain tumors relies on leakage of gadolinium from highly permeable vasculature into the brain parenchyma and thus this process delays its clearance. However, malignant gliomas are highly infiltrative and vascular breakdown is typically not present at the invasive front of the tumor. Therefore, gadolinium-enhanced MRI demonstrates only a part of the tumor that has permeable vasculature. Pirzkall et al. [32] used multivoxel MR spectroscopy to demonstrate the presence of non-enhancing gliomas in areas that has elevated choline signals but no leakage of gadolinium. Similarly, arterial spin labeling can demonstrate regions of malignant gliomas without gadolinium enhancement, which is probably a result of the elevated metabolic demand of the tumor that requires increased blood flow. Furthermore, unlike measurement of the antiangiogenesis effect of mTMZ using cerebral blood volume maps [25], which are calculated values that can be altered by steroid’s effect on vascular permeability, arterial spin labeling has another advantage because it does not require a contrast agent. Instead, this technique utilizes magnetic field gradients and radiofrequency fields to label the endogenous water of blood and, because water is freely diffusible within the brain even without damage to the blood brain barrier, it allows for a quantitative analysis of blood flow in regions that include the malignant glioma [19, 33]. The absolute quantification of blood flow may be limited by regional heterogeneity of the tumor and slight variability may appear in data acquired at different time points. As shown by our data, a blood flow ratio in the tumor normalized to a reference part of the brain may reflect more accurately changes over time.

Alterations in the blood flow ratio have been detected in some of our subjects during treatment with mTMZ. Kerbel et al. [12, 34] demonstrated in an experimental setting that metronomic cyclophosphamide, an alkylator similar to temozolomide but requiring first pass hepatic metabolism to its active agent, delayed or prevented the growth of xenografted tumors in mice. This antitumor effect was most likely mediated by a reduction in the circulating endothelial precursor cells, which are thought to be more sensitive to cytotoxic chemotherapy, and this effect is not specific to cyclophosphamide but also other agents such as cisplatin, vinblastine and vinorelbine [35]. However, in patients with recurrent glioblastomas treated with mTMZ and an antiangiogenic adjuvant celecoxib, immunostaining of CD31-positive endothelial cells of resected tumors before treatment showed high variability in microvessel density [36]. Furthermore, microvessel density did not correlate with patient outcome [36]. Nevertheless, an objective response rate of 5 to 14% and a PFS at 6 months of 17 to 57% were observed in patients treated with mTMZ, suggesting other mechanisms of action may be relevant.

Invasion is a major hallmark of malignant gliomas and antiangiogenesis therapy can bias the tumor towards an invasive phenotype [5, 37, 38]. These invasive glioma cells are thought to possess stem-like cellular characteristics [39]. In this process, MMPs are activated and, in particular, the expression of MMP-2 and MMP-9 is upregulated within the tumor microenvironment [40]. Furthermore, both MMP-2 and MMP-9 activities can also be measured in the CSF, and MMP-9 activity in particular was noted to correlate with disease activity in recurrent glioblastoma [41, 42]. We used activation isoform-specific ELISA as a proxy for MMP-2 and MMP-9 activity within the CSF. In our subjects, the average aMMP-9 level did not correlate with either PFS or OS, but average aMMP-2 level did appear to correlate with OS. This may indicate that the source of aMMP-2, which is constitutively expressed in the brain, may come from sources other than the tumor or the brain parenchyma. Specifically, immune cells can also secrete MMP-2 and MMP-9, and the elevated MMP-2 activity that correlated with OS may indicate an antitumor inflammatory response as a part of innate immunity in the host [43]. However, given the weak correlations between aMMP-2 and aMMP-9 in the CSF and outcomes, it is not clear that mTMZ exerts an anti-angiogenic or anti-invasive effect. In fact, the insignificant changes in metalloproteinases during mTMZ treatment suggest that mTMZ may not work by an anti-angiogenic mechanism and that immunogenic or alkylating effects may have greater relevance.

mTMZ can modulate the immune system to elicit an antitumor response by selective depletion of Tregs [9]. It is notable that at doses given to our subjects that are not cytotoxic to tumor cells, mTMZ still produced a response rate of 14%. This antitumor effect may be the result of Treg depletion that effectively reduces immune suppression within the tumor microenvironment [9, 10, 25–27]. Specifically, Tregs can suppress T lymphocyte activation by inhibiting IL-2 production [44]. Indeed, a high serum level of sIL-2Rα in patients with metastatic melanoma is strongly correlated with poor outcomes from anti-CTLA-4 treatment, which requires concomitant IL-2-mediated immune activation [23]. Likewise, sIL-2Rα modulates IL-2-mediated immune response in patients with follicular lymphoma [24]. Using CSF, we observed that baseline sIL-2Rα was inversely correlated with time to first recurrence of glioblastomas prior to mTMZ treatment, and that the two subjects having elevated baseline levels possessed the longest PFS while the other two with undetectable levels exhibited the shortest PFS. These data suggest a potential contribution of T-cell biology to mTMZ benefit and that patients with elevated CSF sIL-2Rα at baseline have more aggressive disease but they may benefit more from the immunomodulatory effect of mTMZ.

In our cohort, mTMZ was well tolerated and without serious side effects. Although gadolinium enhancement on T1 is observed at the region of the tumor, blood flow as measured by arterial spin labeling showed high variability across individuals and time, with some tumor blood flow increased briefly before subsiding while others showed a gradual decrease or stabilization. The correlation of aMMP-2 with OS and baseline sIL-2Rα with OS both suggest that mTMZ may exhibit a T-cell-dependent immune modulatory effect in patients with recurrent malignant gliomas.

## Conclusion

mTMZ is well tolerated in our cohort with recurrent malignant gliomas. It possesses efficacy against these tumors by altering blood flow, slowing invasion and modulating antitumor immune function.

## Abbreviations

aMMP-2: Activated matrix metalloproteinase-2
aMMP-9: Activated matrix metalloproteinase-9
CI: Confidence interval
CSF: Cerebrospinal fluid
ELISA: Enzyme-linked immunosorbent assay
MMP-2: Matrix metalloproteinase-2
MMP-9: Matrix metalloproteinase-9
MRI: Magnetic resonance imaging
MTIC: 5-(3-dimethyl-1-triazenyl)imidazole-4-carboxamide
mTMZ: Metronomic temozolomide
NK: Natural killer
NKG2D: Natural killer group 2D
OS: Overall survival
PFS: Progression free survival
sIL-2Rα: Soluble interleukin-2 receptor alpha
Tregs: Regulatory T cells

## References

[1] Wong ET, Hess KR, Gleason MJ (1999). Outcomes and prognostic factors in recurrent glioma patients enrolled onto phase II clinical trials. J Clin Oncol.

[2] Kreisl TN, Kim L, Moore K (2009). Phase II trial of single-agent bevacizumab followed by bevacizumab plus irinotecan at tumor progression in recurrent glioblastoma. J Clin Oncol.

[3] Friedman HS, Prados MD, Wen PY (2009). Bevacizumab alone and in combination with irinotecan in recurrent glioblastoma. J Clin Oncol.

[4] Stupp R, Wong ET, Kanner AA (2012). NovoTTF-100A versus physician’s choice chemotherapy in recurrent glioblastoma: a randomised phase III trial of a novel treatment modality. Eur J Cancer.

[5] Iwamoto FM, Abrey LE, Beal K (2009). Patterns of relapse and prognosis after bevacizumab failure in recurrent glioblastoma. Neurology.

[6] Yung WK, Prados MD, Yaya-Tur R (1999). Multicenter phase II trial of temozolomide in patients with anaplastic astrocytoma or anaplastic oligoastrocytoma at first relapse. Temodal Brain Tumor Group. J Clin Oncol.

[7] Yung WK, Albright RE, Olson J (2000). A phase II study of temozolomide vs. procarbazine in patients with glioblastoma multiforme at first relapse. Br J Cancer.

[8] Newlands ES, Blackledge GR, Slack JA (1992). Phase I trial of temozolomide (CCRG 81045: M&B 39831: NSC 362856). Br J Cancer.

[9] Banissi C, Ghiringhelli F, Chen L, Carpentier AF (2009). Treg depletion with a low-dose metronomic temozolomide regimen in a rat glioma model. Cancer Immunol Immunother.

[10] Humphries W, Wei J, Sampson JH, Heimberger AB (2010). The role of tregs in glioma-mediated immunosuppression: potential target for intervention. Neurosurg Clin N Am.

[11] Emmenegger U, Man S, Shaked Y (2004). A comparative analysis of low-dose metronomic cyclophosphamide reveals absent or low-grade toxicity on tissues highly sensitive to the toxic effects of maximum tolerated dose regimens. Cancer Res.

[12] Kerbel RS, Kamen BA (2004). The anti-angiogenic basis of metronomic chemotherapy. Nat Rev Cancer.

[13] Ghiringhelli F, Larmonier N, Schmitt E (2004). CD4 + CD25+ regulatory T cells suppress tumor immunity but are sensitive to cyclophosphamide which allows immunotherapy of established tumors to be curative. Eur J Immunol.

[14] Motoyoshi Y, Kaminoda K, Saitoh O (2006). Different mechanisms for anti-tumor effects of low- and high-dose cyclophosphamide. Oncol Rep.

[15] North RJ (1982). Cyclophosphamide-facilitated adoptive immunotherapy of an established tumor depends on elimination of tumor-induced suppressor T cells. J Exp Med.

[16] Hervieu A, Rebe C, Vegran F (2013). Dacarbazine-mediated upregulation of NKG2D ligands on tumor cells activates NK and CD8 T cells and restrains melanoma growth. J Invest Dermatol.

[17] Lotze MT, Tracey KJ (2005). High-mobility group box 1 protein (HMGB1): nuclear weapon in the immune arsenal. Nat Rev Immunol.

[18] Macdonald DR, Cascino TL, Schold SC, Cairncross JG (1990). Response criteria for phase II studies of supratentorial malignant glioma. J Clin Oncol.

[19] Dai W, Garcia D, De Bazelaire C, Alsop DC (2008). Continuous flow-driven inversion for arterial spin labeling using pulsed radio frequency and gradient fields. Magn Reson Med.

[20] Jarnum H, Steffensen EG, Knutsson L (2010). Perfusion MRI of brain tumours: a comparative study of pseudo-continuous arterial spin labelling and dynamic susceptibility contrast imaging. Neuroradiology.

[21] Kaplan EL, Meier P (1958). Nonparametric estimation from incomplete observation. J Am Stat Assoc.

[22] Murakami S (2004). Soluble interleukin-2 receptor in cancer. Front Biosci.

[23] Hannani D, Vetizou M, Enot D (2015). Anticancer immunotherapy by CTLA-4 blockade: obligatory contribution of IL-2 receptors and negative prognostic impact of soluble CD25. Cell Res.

[24] Yang ZZ, Grote DM, Ziesmer SC (2011). Soluble IL-2Ralpha facilitates IL-2-mediated immune responses and predicts reduced survival in follicular B-cell non-Hodgkin lymphoma. Blood.

[25] Kong DS, Lee JI, Kim JH (2010). Phase II trial of low-dose continuous (metronomic) treatment of temozolomide for recurrent glioblastoma. Neuro Oncol.

[26] Perry JR, Rizek P, Cashman R, Morrison M, Morrison T (2008). Temozolomide rechallenge in recurrent malignant glioma by using a continuous temozolomide schedule: the “rescue” approach. Cancer.

[27] Omuro A, Chan TA, Abrey LE (2013). Phase II trial of continuous low-dose temozolomide for patients with recurrent malignant glioma. Neuro Oncol.

[28] Fulton D, Urtasun R, Forsyth P (1996). Phase II study of prolonged oral therapy with etoposide (VP16) for patients with recurrent malignant glioma. J Neurooncol.

[29] Carney DN (1991). The pharmacology of intravenous and oral etoposide. Cancer.

[30] Blum JL, Dieras V, Lo Russo PM (2001). Multicenter, Phase II study of capecitabine in taxane-pretreated metastatic breast carcinoma patients. Cancer.

[31] Cassidy J, Twelves C, Van Cutsem E (2002). First-line oral capecitabine therapy in metastatic colorectal cancer: a favorable safety profile compared with intravenous 5-fluorouracil/leucovorin. Ann Oncol.

[32] Pirzkall A, Mcknight TR, Graves EE (2001). MR-spectroscopy guided target delineation for high-grade gliomas. Int J Radiat Oncol Biol Phys.

[33] White CM, Pope WB, Zaw T (2014). Regional and voxel-wise comparisons of blood flow measurements between dynamic susceptibility contrast magnetic resonance imaging (DSC-MRI) and arterial spin labeling (ASL) in brain tumors. J Neuroimaging.

[34] Man S, Bocci G, Francia G (2002). Antitumor effects in mice of low-dose (metronomic) cyclophosphamide administered continuously through the drinking water. Cancer Res.

[35] Shaked Y, Emmenegger U, Man S (2005). Optimal biologic dose of metronomic chemotherapy regimens is associated with maximum antiangiogenic activity. Blood.

[36] Stockhammer F, Misch M, Koch A (2010). Continuous low-dose temozolomide and celecoxib in recurrent glioblastoma. J Neurooncol.

[37] Wong ET (2006). Tumor growth, invasion, and angiogenesis in malignant gliomas. J Neurooncol.

[38] Paez-Ribes M, Allen E, Hudock J (2009). Antiangiogenic therapy elicits malignant progression of tumors to increased local invasion and distant metastasis. Cancer Cell.

[39] Sakariassen PO, Prestegarden L, Wang J (2006). Angiogenesis-independent tumor growth mediated by stem-like cancer cells. Proc Natl Acad Sci U S A.

[40] Wang M, Wang T, Liu S, Yoshida D, Teramoto A (2003). The expression of matrix metalloproteinase-2 and −9 in human gliomas of different pathological grades. Brain Tumor Pathol.

[41] Wong ET, Alsop D, Lee D (2008). Cerebrospinal fluid matrix metalloproteinase-9 increases during treatment of recurrent malignant gliomas. Cerebrospinal Fluid Res.

[42] Friedberg MH, Glantz MJ, Klempner MS, Cole BF, Perides G (1998). Specific matrix metalloproteinase profiles in the cerebrospinal fluid correlated with the presence of malignant astrocytomas, brain metastases, and carcinomatous meningitis. Cancer.

[43] Parks WC, Wilson CL, Lopez-Boado YS (2004). Matrix metalloproteinases as modulators of inflammation and innate immunity. Nat Rev Immunol.

[44] Thornton AM, Shevach EM (1998). CD4 + CD25+ immunoregulatory T cells suppress polyclonal T cell activation in vitro by inhibiting interleukin 2 production. J Exp Med.

